# Case report: Transcatheter tricuspid valve-in-valve implantation using novel balloon-expandable aortic valve with 1 year follow-up

**DOI:** 10.3389/fcvm.2023.1152280

**Published:** 2023-07-04

**Authors:** Abdurashid Mussayev, Serik Alimbayev, Nursultan Tanaliev, Aidyn Kuanyshbek, Aripov Marat, Timur Lesbekov, Yerkezhan Raissov, Aigerim Sadykova, Askarovna Kenzhebayeva Kamila, Murat Mukarov, Yuriy Pya

**Affiliations:** ^1^ Head of Cathlab, National Research Cardiac Surgery Center, Astana, Kazakhstan; ^2^ Head of Structural Heart Diseases, National Research Cardiac Surgery Center, Astana, Kazakhstan; ^3^ Department of Interventional Cardiology, National Research Cardiac Surgery Center, Astana, Kazakhstan; ^4^ Head of the Department of Anesthesiology, Resuscitation and Intensive Care, National Research Cardiac Surgery Center, Astana, Kazakhstan; ^5^ Head of Department of Interventional Cardiology, National Research Cardiac Surgery Center, Astana, Kazakhstan; ^6^ Head of the Department of Cardiac Surgery, National Research Cardiac Surgery Center, Astana, Kazakhstan; ^7^ Resident of Interventional Cardiology, National Research Cardiac Surgery Center, Astana, Kazakhstan; ^8^ Chief Nurse of Cathlab, National Research Cardiac Surgery Center, Astana, Kazakhstan; ^9^ Clinical Cardiologist, Central Hospital with a Polyclinic of the Ministry of Internal Affairs, Astana, Republic of Kazakhstan; ^10^ Head of the Department of Cardiology, National Research Cardiac Surgery Center, Astana, Kazakhstan; ^11^ Department of Cardiac Surgery, National Research Cardiac Surgery Center, Astana, Kazakhstan

**Keywords:** bioprosthetic heart valve, balloon expandable heart valves, Myval transcatheter heart valve, tricuspid valve, valve-in-valve procedure, valvular heart disease

## Abstract

Generally, the dysfunction or failure of bioprosthetic heart valves (BHVs) is managed by replacement surgery. In the case of tricuspid valve dysfunction, re-do surgery is rarely attempted because of the critically high risk of developing pulmonary hypertension, pulmonary embolism, and intraoperative mortality. Hence, transcatheter tricuspid repair and replacement procedures are preferred. More recently, transcatheter valve-in-valve (ViV) treatments have gained importance because of their less invasiveness, especially for patients with prior surgeries. Encouraging evidence of the safety and effectiveness of a novel balloon-expandable (BE) transcatheter heart valve (THV)—the Myval THV—has been reported for ViV procedures. Here, we present a case-series of 5 patients, in whom tricuspid ViV procedure was performed using BE Myval THV, implanted supra-annularly by anchoring onto the deteriorated BHV. This case-series details the procedural steps to prevent in-hospital adverse events and early (30-day) mortality and the challenges during tricuspid ViV interventions.

## 1. Introduction

Aside from Ebstein’s anomaly, tricuspid valve degeneration or prolapse can occur due to other serious primary and secondary causes, including carcinoid syndrome, infective endocarditis, or Marfan’s syndrome (1). Patients suffering from any of these conditions are required to undergo tricuspid valve replacements, often at a younger age (<60 years) (1). Replacements of the tricuspid valve have been performed using mechanical valves as well as the bioprosthetic heart valves (BHVs), which may gradually deteriorate or dysfunction. The functional durability of a BHV implanted in the tricuspid position is often limited and almost 40% of those the patients needed re-intervention within 5 to 15 years from the initial surgery (2). Isolated tricuspid valve surgery is rarely performed because of the high operative mortality rates, especially for reoperations (3). Moreover, tricuspid BHV dysfunction is associated with increased mortality rates and poor clinical outcomes (3–5). Ever since the first report of a percutaneous transcatheter aortic valve replacement by Cribier et al, there has been increased interest in performing transcatheter tricuspid valve replacements (6), which has widened the treatment options for patients requiring tricuspid replacement and reduced the health economic burden associated with this high-risk group of patients.

Valve-in-valve (ViV) implantation for the treatment of degenerated BHV in the tricuspid position is reported to be feasible with favorable long-term outcomes using a balloon-expandable (BE) transcatheter heart valve (THV) of the Sapien THV series (Edwards Lifesciences, Irvine, CA, USA) (5, 7–9). A newer-generation BE THV that seems potentially useful for transcatheter tricuspid ViV procedures is the Conformité Européenne (CE)*-*marked BE Myval™ THV (Meril Life Sciences Pvt. Ltd., India). The safety and efficacy of this BE THV is well established in patients with severe aortic stenosis (10, 11). A selected few case reports present some promising outcomes data of using the BE Myval THV for tricuspid valve-in-valve treatments (3, 5, 12). Recently, De Costa et al., described the implantation of a 32 mm BE Myval THV in the tricuspid position to replace the previous failed bioprosthetic valve (with approximate 32 mm internal diameter and 29 mm true internal diameter) (3). Similarly, another article reported the use of BE Myval THV for a patient whose previous bioprosthesis (Epic, St. Jude Medical, Saint Paul, Minnesota, US) showed signs of valve failure after 11 years (7).

As there is a dearth of published data on the application of the study device, particularly for tricuspid valve-in-valve treatments, we present a case series of the 5 clinical cases involving the use of BE Myval THV. In these cases, the patients experienced symptoms of tricuspid insufficiency due to the failure of their implanted bioprosthesis, thereby requiring tricuspid ViV replacements. The baseline characteristics of the patients are summarized in Table 1. The pre-operative multi-slice computed tomography was analyzed using the 3mensio Structural Heart 10.3 software by NRCSC and Meril’s TAVI Core Lab. Further, the ViV sizing application was used for appropriate valve selection, (mplsheart.org/viv). We elucidate their individual case summaries and clinical management and describe how the use of ViV size selection software prior to the percutaneous procedure enhanced the procedural success in each case. Written Informed consent was obtained from all patients and Institutional ethical committee approval was obtained prior to procedure.

**Table 1:** Baseline, anatomical and procedural characteristics of patients undergoing tricuspid ViV with BE Myval THV and in-hospital observations.

Case		1	2	3	4	5
Age (Years)		40	40	54	46	71
Gender		F	F	F	F	F
BMI (Kg/m^2^)		28.8	30.85	30.85	17.83	21.46
NYHA class		IV	III	III	III	III
Logistic Euroscore- II		20.96%	5.48%	20.96%	8.70%	16.50%
GFR (ml/min)		64	80	60	87	80
LVEF (%)		45	57	44	51	43
Arterial hypertension		No	No	Yes	No	Yes
Diabetes mellitus		No	No	Yes	No	No
Atrial fibrillation		Yes	Yes	Yes	Yes	Yes
Severe pulmonary hypertension		No	No	No	No	Yes
CAD		No	No	No	No	No
Failed prosthesis type		Pericarbon More	Hancock II	Hancock II	Hancock II	Pericarbon More
Size (mm)		33	31	29	27	29
Years since implant		9	4	8	5	11
Failure mode		TS + TR	TS + TR	TS + TR	TR	TS
Leaflets calcification grade		+	+	+	+	+
True Internal diameter (mm)		29	26	24	22	25
CT annulus area (mm^2^)		665	673	657	438	530
CT annulus perimeter (mm)		92	91	91	73	82
Access site		Transfemoral	Transfemoral	Transfemoral	Transfemoral	Transfemoral
Type of anesthesia		Local/sedation	Local/sedation	Local/sedation	Local/sedation	Local/sedation
Myval size (mm)		29	29	29	24.5	27.5
Transthoracic echocardiography						
Transvalvular gradient P-P\Mean	Pre	23\13	13\6	19\8	10\4	21\13
	Post	7\3	4\2	5\3	6\3	8\4
Valve regurgitation	Pre	Moderate	Moderate	Severe	Severe	Mild
	Post	Trace	None	None	None	Trace
Anatomical and procedural characteristics						
Pre-dilatation		No	No	No	No	Yes
Post-dilatation		No	No	No	No	No
Supporting wire in RV		Lunderquist	Lunderquist	Lunderquist	Lunderquist	Lunderquist
Access site management		Z-stitch	Z-stitch	Z-stitch	Z-stitch	Z-stitch
Fluoroscopy time (mins)		13.2	15.7	17	10	58
Contrast media (ml)		20	30	60	24	10
In-Hospital outcomes						
Patient prosthesis mismatch		No	No	No	No	No
Bleeding (BARC)		No	No	No	No	Yes
VARC2 complications		No	No	No	No	No
Permanent pacemaker implantation		No	No	No	No	No
Stroke/TIA		No	No	No	No	No
Peri-procedural MI		No	No	No	No	No
Hospitalization length		13	8	4	12	9

F, Female; BMI, Body mass index; NYHA, New York Heart Association; GFR, Glomerular filtration rate; LVEF, left Ventricular ejection fraction; CAD, Coronary artery disease; TS, Tricuspid stenosis; TR, Tricuspid regurgitation; CT, Computed Tomography; TIA, Transient ischemic attack; MI, Myocardial Infarction.

## 2. Case presentation

### 2.1. Case 1

**Figure 1:**
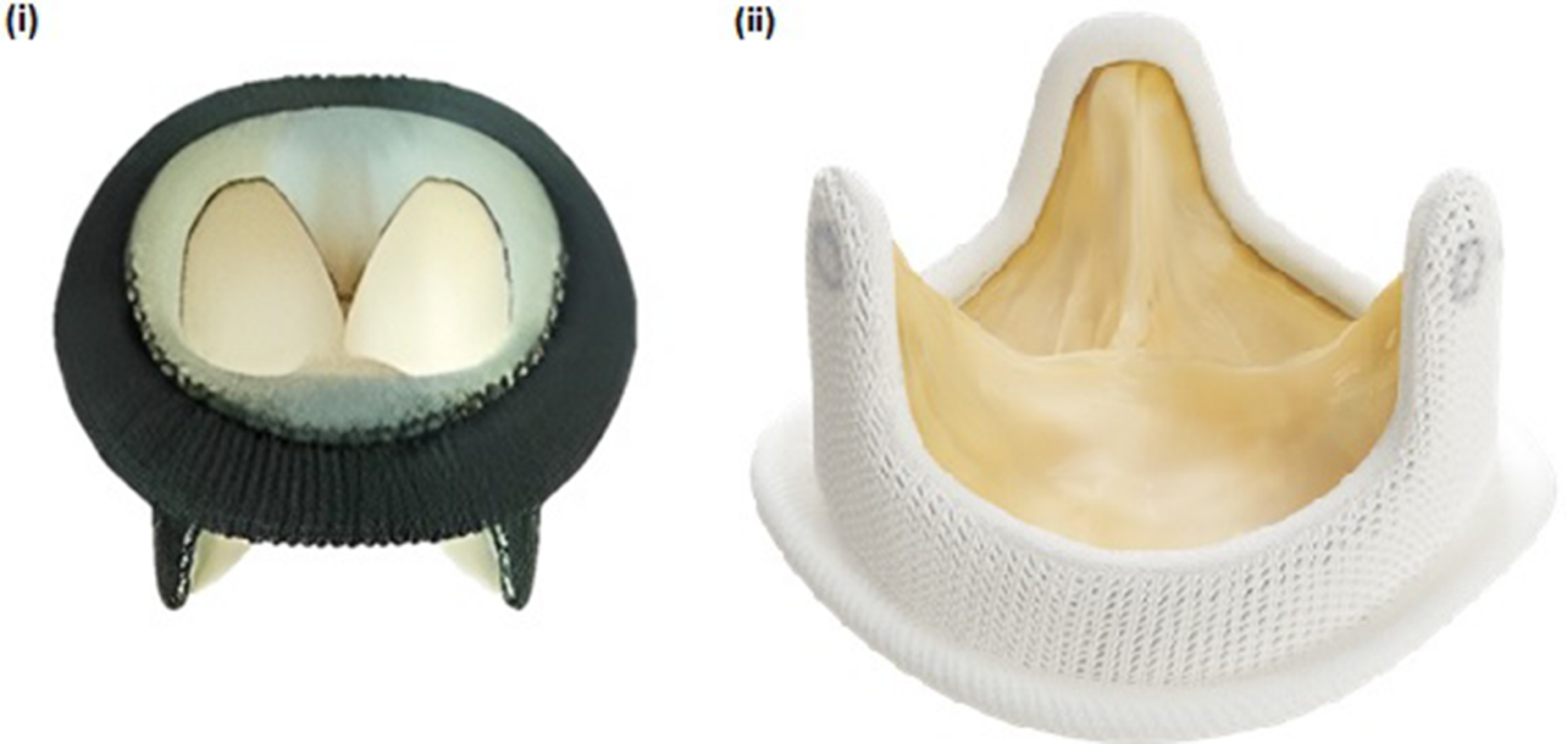
(i) pericarbon MORE surgical bioprosthetic valve (ii) hancock II surgical bioprosthetic valve.

**Figure 2:**
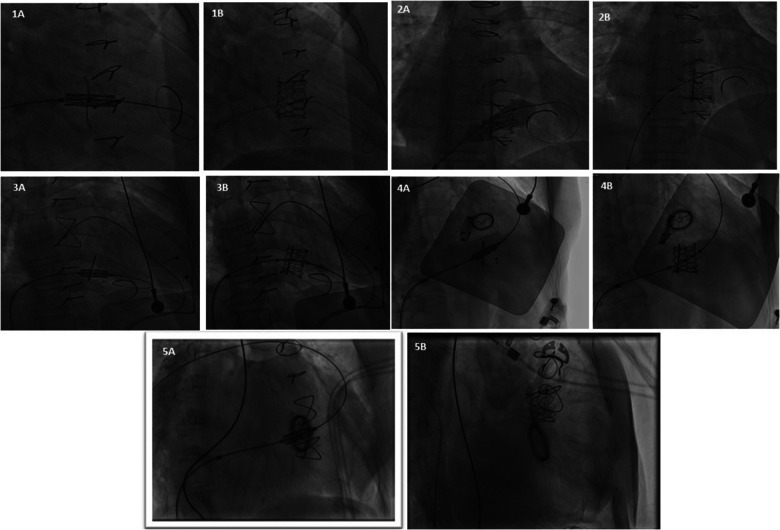
Represents fluoroscopic imaging after VIV implantation of all 5 patients (case 1 to case 5).

A 40-year-old female presented to our center with atrial fibrillation, symptomatic congestive heart failure, New York Heart Association (NYHA) functional class IV and tricuspid regurgitation due to a failed 33 mm Pericarbon More™ (Sorin Group, Italy) BHV [Figure 1(i)] surgically replaced 9 years ago (Ebstein’s anomaly) as a surgical indication. A 29 mm BE Myval THV (−0.5% downsizing) with 2 ml mixed contrast + saline was chosen and successfully implanted under local anesthesia under fluoroscopic guidance (Table 1, Figures 2: 1A,B). No pacing was required for the inflation of BE THV. No significant paravalvular leak (PVL) nor conduction disturbances were reported. The patient was discharged 7 days after the procedure on oral anticoagulant therapy: Vitamin K antagonists (VKA). Clinical and echocardiographic results at 6 months and 1-year are reported in Table 2.

**Table 2:** Clinical follow-up of 6 months and 1-year following tricuspid ViV implantation with BE Myval THV.

Case	1		2		3		4		5	
	6 Months	12 months	6 Months	12 months	6 Months	12 months	6 Months	12 months	6 Months	12 months*
NYHA FC	I		II		II		II	I	III	NA
MI	No		No		No		No		No	NA
Stroke\TIA	No		No		No		No		No	NA
Valve failure	No		No		No		No		No	NA
Transvalvular gradient P-P\Mean	6\4	7/4	4\2	4.3	5\2	5/3	6\4		7\3	NA
Valve regurgitation grade (0–4)	None	1	None	1	None	0	None	0	None	NA

NYHA FC, New York Heart Association functional class; TIA, Transient ischemic attack; MI, Myocardial Infarction; P-P, Peak pressure gradient.

* Case 5: At 12 months follow-up patient was reported dead.

### 2.2. Case 2

A 49-year-old female with prior tricuspid valve replacement (8 years back) with a surgical bioprosthesis Hancock II 31 mm [Figure 1(ii)] (associated with Glenn’s procedure because of Ebstein’s anomaly) was admitted to our hospital. Her presenting symptoms were exertional dyspnea (NYHA III) and atrial flutter. On her echocardiogram, a degenerated BHV with mixed tricuspid valve disease (stenosis and regurgitation) was revealed. Although the patient was young, the local heart team decided for transcatheter ViV implantation. A 29 mm Myval THV (−1.7% of downsizing) plus 3 ml mixed contrast + saline was successfully implanted (no trans-valvular gradient, PVL or conduction disturbances) within the degenerated BHV [Table 1, Figures 2: 2A,B]. Pacing was not performed for the deployment of BE THV. A day before transcatheter tricuspid ViV implantation, successful transcatheter radiofrequency ablation of the atrial flutter was performed (Table 1). The patient was discharged after 7 days with oral anticoagulant therapy (VKA). At 6 months and 1-year follow-up, symptoms significantly improved with no echocardiographic signs of ViV deterioration (Table 2).

### 2.3. Case 3

A 55-year-old female with Ebstein’s anomaly underwent surgical tricuspid valve replacement with a St. Jude Medical Biocor 31 mm BHV with pacemaker implantation due to post-operative complete atrio-ventricular block. Two years later, the patient required a re-do surgery with Hancock II 29 mm BHV [Figure 1(ii)] (Biocor degeneration with stenosis and regurgitation) plus Glenn’s procedure and the explant of infected pacemaker leads. Finally, epicardial electrodes were implanted as well as PPM lead into the anterior abdominal wall on the left side. The procedure was performed under venous-arterial ECMO support.

Eight years later, the patient was admitted with complaints of exertional dyspnea (NYHA III) and bilateral limb oedema. Transthoracic echocardiography revealed a severe degeneration of the surgical BHV. The institute’s heart team decided to proceed with ViV procedure. For this patient, a 29 mm Myval THV (0.1% of oversizing) was chosen and effectively implanted, without pacing (Table 1, Figures 2: 3A,B). The patient was discharged on oral anticoagulant therapy (VKA) 3 days later with good outcomes for up to 1 year (Table 2).

### 2.4. Case 4

A 48-year-old female with diseased aortic and mitral valves (rheumatic etiology) underwent replacement of the respective valves with mechanical heart valves 16 years ago. Some years later, the patient developed symptomatic, severe tricuspid regurgitation with signs of congestive heart failure. Re-do surgery was required and a Hancock II 27 mm BHV was implanted [Figure 1(ii)] at the tricuspid position. Five years later, the patient was admitted to our center because of atrial fibrillation and exertional dyspnea (NYHA III). A degenerated BHV with severe regurgitation was detected and the patient was recommended for a ViV procedure. According to the BHV size measurements, a 24.5 mm BE Myval THV (7.1% degree of oversizing) was chosen for this young patient. The transcatheter tricuspid ViV implantation was effectively performed under fluoroscopic guidance (Table 1, Figures 2: 4A,B) without pacing. No major complications occurred during hospitalization and the patient was discharged after 4 days on oral anticoagulant therapy (VKA). At 1-year follow-up NYHA class improved while no PVL or significant gradient was noted in the echocardiography report (Table 2).

### 2.5. Case 5

A 70-year-old female with severe, symptomatic mitral stenosis (rheumatic etiology) underwent surgical commissurotomy. Almost 17 years later, the patient developed symptomatic mitral stenosis and tricuspid regurgitation requiring surgical replacement of both valves {mechanical prosthesis Carbomedics 27 mm in mitral position and Pericarbon More 29 mm in tricuspid position [Figure 1(ii)]. Some years later, a stenotic degeneration of the tricuspid BHV occurred. The patient was referred for ViV procedure and a 27.5 mm BE Myval THV (12.5% degree of oversizing) was chosen. After balloon pre-dilatation with a 25 × 40 mm Mammoth balloon (Meril Life Sciences Pvt. Ltd., India), the BE Myval THV was successfully implanted, without pacing (Table 1, Figures 2: 5A,B) but three hours after the procedure, due to moderate femoral vein bleeding, two units of packed red blood cells were transfused, and surgical intervention was needed to manage this complication. Seven days after the procedure, the patient was discharged on anticoagulant therapy (VKA) (Table 2). During the 1-year follow-up, it was reported that the patient had died (Table 2).

However, we noted the improvement in the conditions of the other four patients, without any signs of THV deterioration for 1 year follow-up duration as summarized in Table 2.

## 3. Discussion

This case-series demonstrates the successful implantation of the BE Myval THV in a group of patients with prior valve replacements and concomitant surgery. This group of patients with prior valve surgery are at higher risks during reoperation, especially involving tricuspid valve replacement surgery. To manage the high patient burden associated with tricuspid valve pathologies indicated for re-do surgery, transcatheter valve-in-valve replacements have been suggested (3). Favorable results have been demonstrated with both BE THV and a self-expandable THV for ViV or valve-in-ring interventions (5, 12, 13). Encouraging results have been reported following implantation of a BE THV such as SAPIEN series and Myval THV in the aortic, mitral, and tricuspid positions (14–18).

Recently, BE Myval THV has been shown to be safe and effective at par with BE Sapien 3 and SE Evolut R THVs (10). In particular, an immediate post-procedural improvement in the hemodynamic performance and a lower risk of conduction disturbances have been shown vs. Sapien 3 (10) and lower rate of significant (more than moderate) PVL vs. Evolut R following implantation in a native diseased aortic valve (11). Moscarella et al., conducted a multicenter observational study of 267 aortic and mitral ViV and valve-in-ring procedures, in which the VARC-3 post-procedural technical success rate was evaluated to be 98%. Furthermore, no cases of BHV failure were noted, despite a high rate of valvuloplasty performed prior to THV deployment in the mitral positions (18). In this case-series, the BE Myval THV was used on an off-label basis. Given its acceptable outcomes reported in contemporary studies on ViV procedures in the aortic and mitral positions (19), we believe that the BE Myval THV may show high suitability for tricuspid ViV procedures as well.

There is limited evidence on the hemodynamic performance and technical safety of BE Myval THV implantation in a degenerated BHV (7, 8) in the tricuspid ViV setting. Hence, this case-series presents crucial real-world data of our experience with this device. As stated above, we report procedural and device success in all 5 cases without vascular bleeding, conduction disturbances, or paravalvular leakage in a very high-risk group of patients.

A valve-in-valve size selection software is useful for predicting or verifying the correct valve size, orientation, and the accurate placement of a BE THV (20). Avoiding malapposition, embolization, and coronary obstruction remain key factors related to the success of a ViV intervention. In this case series, a smartphone-app has been used that predicts the ideal implant ViV implant position and the true internal annulus diameter, (MHIF ViV Digital Application, Minneapolis Heart Research Institute, Minneapolis, MN, USA) (20). The estimation of the true internal diameter of the existing bioprosthetic valve implant is key to achieving a successful ViV implantation. In our case-series, the mean oversizing was 12% for all 5 cases, indicating a good fit.

The optimal performance of this novel BE Myval THV could be related to the wider range of suitable THV sizes compared to other commercially available THVs (21). In particular, the intermediate sizes reduce the risks of both oversize or incomplete ViV “sealing” that can be associated with (i) free wall rupture, or (ii) significant PVL or premature THV dysfunction (18). Although tricuspid ViV procedure could be performed through the trans-jugular vein approach, we report cases through the trans-femoral route, and only one case was complicated by bleeding requiring surgery. Despite this, we strongly believe that ViV procedures using the BE THV represents a paradigm shift in the approach to treat failed tricuspid valve prostheses. Herein, we demonstrate a safe and effective procedure with BE Myval THV suitable for very high-risk patients with encouraging outcomes data. These data provide a preliminary basis for other investigators to investigate the long-term outcomes of ViV procedures in the tricuspid position and compare the outcomes with those of the CE and Food and Drug Administration (FDA)-approved ViV devices such as SAPIEN XT (Edwards Lifesciences, Irvine, CA, USA) and CoreValve (Medtronic Inc., Minneapolis, MN, USA) (20).

### 3.1. Limitations

Despite reporting crucial primary data on the off-label use of a novel BE THV for tricuspid ViV procedures with successful implantation outcomes up to one year, our study is not without limitations. In particular, we note that one of the main contraindications for TVIV implantation is infective endocarditis. Secondly, the case series is limited by the small number of patients. Since this is a preliminary report of rare cases requiring tricuspid ViV procedures, the patient selection criteria for the intervention needs attention. As such, there are no size-related boundaries to be considered while implanting the BE Myval THV in the tricuspid position. Furthermore, we believe that the reported information of the transcatheter tricuspid ViV procedures through the transfemoral approach in a selected group of high-risk patients having undergone prior multiple surgeries is instrumental to assess the feasibility of implanting the novel THV in the tricuspid position without pacing. This case series is beneficial for gathering knowledge on preventing coronary obstruction while performing transfemoral transcatheter tricuspid ViV procedures. Future investigations with a long-term follow-up would help estimate the durability of the novel BE THV in the same indication for larger populations and assess the clinical outcomes. Lastly, the data of this case-series would encourage other interventional cardiologists for considering the clinical use of the BE Myval THV in selected cases of failed tricuspid procedures with appropriate anatomical feasibility.

### 3.2. Future directions

The case series includes 5 unique cases of high-risk patients with prior surgeries, in whom tricuspid ViV procedures were clinically indicated and performed after careful ViV size selection. The reported data is crucial for analyzing the potential feasibility of the BE Myval THV in the setting of tricuspid ViV implantation. The incidence of failed bioprosthetic heart valves is deemed to increase in the forthcoming years led by the increase in the number of patients receiving surgical valve replacement instead of palliative medical therapy or undergoing surgical repair. Hence, the small cohort of 5 cases may guide the assessment of the clinical outcomes for larger multicenter cohorts with recurrent tricuspid valve disease, in whom treatment options are limited due to anatomical challenges and the critically limited choices of device selection. As the need for exploring transcatheter tricuspid ViV procedures expands, a wider array of BE THVs would be necessary to cater to the different demographics in respect to small or large annulus diameters, which currently is an unmet clinical need. In this regard, the preliminary data from this case series with the BE Myval THV are encouraging and may be used to treat geriatric patients with a high surgical risk as well as younger patients with valvular heart disease following the failure of implanted bioprosthetic valves. The routine clinical cardiology practice may benefit from the case-specific technicalities of this case-series in preventing coronary obstruction and avoiding the need for right ventricular pacing in similar cases of high complexity, which would help identify the case selection criteria for transcatheter tricuspid ViV procedures in future.

## 4. Conclusion

Transcatheter tricuspid ViV implantation using the BE Myval THV resulted in feasible and safe with good immediate and 1-year clinical outcomes. Further investigations with a larger cohort of patients and longer-term follow-up are needed to confirm our initial encouraging experience.

## Data Availability

The original contributions presented in the study are included in the article, further inquiries can be directed to the corresponding author.
